# The case for global investment in rheumatic heart-disease control

**DOI:** 10.2471/BLT.13.134486

**Published:** 2014-09-08

**Authors:** Rosemary Wyber, Liesl Zühlke, Jonathan Carapetis

**Affiliations:** a Telethon Kids Institute, University of Western Australia, 100 Roberts Road, Subiaco, Western Australia, 6008, Australia.; b Departments of Paediatrics and Medicine, University of Cape Town, Cape Town, South Africa.

Rheumatic heart disease comprises a small proportion of the total global disease burden according to current estimates. A rare complication of a streptococcal throat infection, rheumatic heart disease causes heart valve damage and progressive heart failure. The cause and course of this disease can be difficult to explain to policy-makers and to people at risk. The relative burden and complexity of the disease have contributed to its neglect by governments, donors and decision-makers. We argue that the World Health Organization (WHO) and national governments should rekindle their rheumatic heart disease control programmes.

Rheumatic heart disease is now unusual in most high-resource settings because of access to health care and availability of antibiotics. However, it remains endemic in socioeconomically vulnerable populations in high-income countries and in low- and middle-income country settings.^1^ Prevention and control measures for rheumatic heart disease include reduction of household crowding, timely diagnosis and appropriate antibiotics for bacterial pharyngitis and – in people who develop rheumatic fever – antibiotic prophylaxis over several years to prevent disease progression. 

Global public health has no shortage of challenges such as improving sanitation, eradicating polio and preventing tobacco use. A utilitarian approach pervades attempts to deliver the best possible health care for the greatest number of people. Limited human, financial and logistical resources make prioritization essential. Funding and policy meetings are increasingly focused on identifying easily achievable and high impact global health interventions. However, only a fraction of global health needs are amenable to simple and scalable interventions. When and why should time, energy and money be invested in more complex problems? Reflecting on these uncertainties, we build the case for investing in global control of rheumatic heart disease, with a focus on highly endemic settings.

## Existing knowledge

Research is still needed on the causes, diagnostic methods, and clinical management of rheumatic heart disease.^2^^,^^3^ However, the basic framework for disease control has existed since the 1950s, with incremental improvements in the structure of control programmes and the ways in which these are delivered.^4^ The usefulness of comprehensive disease-control programmes has been demonstrated by local or state programmes in Australia, Cuba, Guadeloupe, Martinique, New Zealand and the United States of America.^4^ These data show that rheumatic heart disease is a preventable, noncommunicable cardiovascular disease acquired in childhood. Early and effective intervention can avert premature cardiovascular mortality in these patients. At a time when there is an increased focus on averting premature cardiovascular mortality, rheumatic heart disease exemplifies a condition amenable to early and effective intervention.

## Underestimated disease burden

The benchmark estimates of the rheumatic heart disease burden are based on a 2005 review encompassing 57 studies. This global review estimates 15.6 million prevalent cases, 282 000 incident cases and 233 000 deaths annually. However, a shortage of reliable epidemiological data has been widely acknowledged and the true burden of the disease is expected to be far higher than the benchmark estimates.^1^^,^^5^ Despite likely underestimates, the global burden of disease study calculated a disability-adjusted life year (DALY) burden of 1430 (range: 944–2067) in 2010 – approximately one quarter of the global DALY burden of cancer.^5^^,^^6^ The significance of subclinical rheumatic heart disease and the potentially fatal sequelae of rheumatic heart disease (congestive heart failure, endocarditis, atrial fibrillation and stroke) remain under-explored.^5^ The current benchmark provides a conservative estimate for rheumatic heart disease prevalence; the true number of people living with rheumatic heart disease is likely to be higher and in the coming years these estimates will be adjusted.

## Indicator of inequality

Sustained control of rheumatic heart disease at a population level demands a high-functioning health system that meets the needs of vulnerable people. In high-income settings, rheumatic heart disease demonstrates persistent inequality. For example, indigenous Australians in the Northern Territory under the age of 35 years are 122 times more likely to have rheumatic heart disease than their non-indigenous peers in the same region.^7^

This pattern of inequality by socioeconomic and indigenous status is seen worldwide. Reduced economic participation, premature mortality and maternal mortality contribute to sustained poverty in these groups for generations to come. Rheumatic heart disease offers a barometer of health-care delivery and inequality. Its role as an indicator of a functioning health system was illustrated by the surge in cases of acute rheumatic fever in the aftermath of the dissolution of the former Union of Soviet Social Republics in central Asia.^8^

## The disease community

The rheumatic heart disease community is a relatively small entity of a few hundred clinicians, researchers and advocates.^3^ Compared with the vast array of stakeholders in large disease communities – such as tuberculosis and malaria – a smaller community may profit from simplicity and decreased costs. In addition, a small network of committed stakeholders yields efficiency gains in communication and cohesion, providing an opportunity to identify and implement a strategic plan for global disease control.

## Clinical engagement

Clinicians on the front line of health-care delivery in low-resource settings respond more to clinical need than to global health priority-setting frameworks. The persistent emergence of rheumatic heart disease initiatives indicates a clinical demand that is inadequately captured in global burden of disease estimates and priority setting frameworks. Rheumatic heart disease can cause progressive disability and death in early adulthood. Pregnancy and labour are particularly risky for women with rheumatic heart disease, contributing to maternal mortality in low-resource settings.^9^ The consequences of rheumatic heart disease in highly-endemic settings has inspired research projects, cardiac surgery programmes and the creation of support groups.^3^ Without sufficient funding, these important initiatives will achieve only local impact.

## Cost–effectiveness

Heart failure in young people living with rheumatic heart disease motivates considerable investment in end-stage treatment. A recent survey identified 80 humanitarian organizations that provide paediatric cardiac surgery in resource-limited settings.^10^ Governments spend sizeable proportions of health budgets on international care. For example, in Samoa, 12% of the overseas treatment budget was spent on international surgery for people with rheumatic heart disease.^11^ In many countries affected individuals and families are forced to go into debt or attempt fundraising.^2^ However, surgery is palliative for many patients. The cost of end-stage interventions is economically and socially higher than that of comparatively low-cost comprehensive control programmes with an emphasis on prevention.

## Capitalize on investments

WHO coordinated a Global Rheumatic Heart Disease Control Programme from 1984–2002. By 1990, sixteen countries had disease registers for rheumatic heart disease, 1.5 million school-aged children had been screened for the disease and nearly 25 000 health and education staff had received rheumatic heart disease training.^12^ Although the WHO programme yielded valuable lessons and created networks of disease-control experts, competing health priorities diverted funding in the early 2000s.^13^ The World Heart Federation has successfully maintained some of these networks. However, the opportunity to capitalize on components of the WHO programme will diminish with time and the cost of launching new initiatives in the future will be much higher. 

## Diagonal health-care delivery

Rheumatic heart disease intersects with several disease communities: infectious diseases, noncommunicable diseases, neglected tropical diseases and childhood diseases. Control programmes require partnerships with those working on access to medicines, global surgery initiatives and notifiable disease systems. Rheumatic heart disease necessitates and exemplifies a diagonal approach from robust primary to highly specialized tertiary care.

## A neglected disease

Acute rheumatic fever and rheumatic heart disease are neglected by governments, civil society, patient advocates and funding agencies. In contrast, an identifiable community has formed around neglected tropical diseases and has successfully mobilized resources and developed control strategies. However, acute rheumatic fever research attracted only 0.01% of funding for neglected diseases between 2007 and 2011.^14^ Funding for epidemiologic surveillance and disease-control interventions is thought to be even less. We have no information about current levels of funding for rheumatic heart disease research.

Twenty years ago, a review appraising approaches to rheumatic heart disease control noted: “In the current era of primary health care, vertical programs for the control of specific diseases such as rheumatic heart disease are in disfavour.”^15^ The review built a case for extending simple and cost–effective measures to all countries. Had these recommendations been put into action, significant progress could have already been made. Another twenty years of relative stasis is unconscionable; particularly if intervention is delayed because rheumatic heart disease does not fit with the increasingly rigid demands of global health funding or programming.

The World Heart Federation has a goal to reduce premature deaths from rheumatic fever and rheumatic heart disease among individuals aged less than 25 years by 2025.^13^ To achieve this target globally, nationally and locally, a roadmap is needed. There are strong pragmatic and humanitarian reasons for investing in measures to reduce the prevalence and premature mortality of rheumatic heart disease.
